# Parent-focused online intervention to promote parents’ physical literacy and support children’s physical activity: results from a quasi-experimental trial

**DOI:** 10.1186/s12889-022-13739-z

**Published:** 2022-07-12

**Authors:** Amy S. Ha, Qing He, David R. Lubans, Cecilia H. Chan, Johan Y. Y. Ng

**Affiliations:** 1 Department of Sports Science and Physical Education, Faculty of Education, The Chinese University of Hong Kong, Hong Kong, China; 2 Bau Institute of Medical and Health Sciences Education, Faculty of Medicine, The University of Hong Kong, Hong Kong, China; 3 Priority Research Centre for Physical Activity and Nutrition, School of Education, University of Newcastle, Callaghan, NSW Australia

**Keywords:** Parent-focused intervention, Physical literacy, Primary school children, Moderate-to-vigorous physical activity

## Abstract

**Background:**

The development of physical literacy (PL) early in life may influence children's subsequent physical activity (PA) participation and consequent health benefits across the life course. Interventions designed for parents are lacking, but such efforts can potentially enhance the PL of parents and their children’s PA participation. Additionally, there is insufficient evidence to support the feasibility of delivering a PL intervention using an online format. Therefore, the purpose of this study was to examine the feasibility and effectiveness of a parent-focused, theory-driven, online-delivered intervention designed to improve the parents’ PL and children’s PA behaviors.

**Methods:**

A non-randomized trial was conducted to evaluate the effects of the program. 224 Hong Kong families (primary school-aged children and their parents) registered to the program were considered the experimental group and were exposed to an online intervention over three months. Another 220 families in Hong Kong were considered the comparison group and did not receive any intervention. Outcome measures included PA behaviors (daily steps and moderate-to-vigorous PA), parent–child co-activity behaviors, family PA routines, and parent perceived PL. Linear mixed models were used to analyze the differences in terms of changes in measured outcomes between groups over time.

**Results:**

No significant group-by-time effects were found for children’s or parents’ PA behaviors. In terms of the family Co-PA routines, a small positive effect size in favor of the experimental group was found (*p* = .44, *d* = 0.2). Group-by-time effects favorable to the experimental group was detected for parent–child co-activity (*p* < .001, *d* = 0.7) and parental PL (*p* < .001, *d* = 0.9) at post-intervention. The results demonstrated that the intervention was acceptable and that there was potential for scale up.

**Conclusions:**

Findings indicated that the intervention was effective in increasing parent–child co-activity and parent perceived PL. During the pandemic, online intervention delivery was found to be feasible. Using this mode of delivery, the intervention has the potential to reach a wide population in the local context.

**Trial registration:**

The study was prospectively registered at the Chinese Clinical Trial Registry, Registration number: ChiCTR2100041903, Registered 09 January 2021.

## Background

Regular participation in physical activity (PA) contributes to physical health development, enhances physical fitness, and reduces the risk of chronic diseases [1]. PA also plays a preventive role in childhood obesity [2, 3]. Furthermore, maintaining an active lifestyle decreases the incidence of mental health disorders, such as anxiety and depression, and increases self-esteem and cognitive functioning outcomes [4]. Developing a healthy lifestyle during childhood increases the possibility of carrying over such patterns later in life [5]. However, the prevalence of children participating in sufficient PA is low in Hong Kong [6]. Ha and Ng [7] measured primary school children’s PA levels using accelerometers and found only 6.1% of children met the 60 min moderate-to-vigorous physical activity (MVPA) per day guideline proposed by the World Health Organization. These findings suggest there is a pressing need to intervene and enhance children’s PA behaviors.

### Physical literacy

The promotion of physical literacy (PL) offers a potentially promising approach for childhood PA interventions [8]. The concept of PL is emphasized as an important ideology and defined as *“the motivation, confidence, physical competence, knowledge and understanding to value and take responsibility for engagement in physical activities for life”* [9]. There are still some debates in terms of how PL should be defined, but the notion of moving from a unidimensional construct of PA to a more holistic one is agreed upon by many researchers [10]. Even though there might be different clear-cut definitions of PL, Whitehead’s framework [9] is broadly used (i.e., 70% of papers adopted the concept) and widely accepted by most people. Moreover, compared with other PL concepts, Whitehead’s concept of PL is based on the premise of a holistic, individualized journey [10]. Cairney et al. [8] proposed a PL conceptual model and stated that the development of PL early in life may influence their subsequent PA participation and consequent health benefits across the life course [8, 10]. PL is considered a dynamic concept, which consists of four domains: affective domain (motivation and competence), physical domain (physical competence), cognitive domain (knowledge and understanding), and behavioral domain (engagement in physical activities for life) [11]. Taken together, these four domains represent a holistic approach to lifelong PA participation [12]. PL may offer a more holistic and reflexive approach to incorporating PA promotion into the behavioral change and healthcare setting, where context, psychosocial aspects, individual competence, and understanding and knowledge may be concerned [13].

### The role of parents and parent-focused intervention

Being primary caregivers, parents typically play an essential role in children's growth and development and in shaping their PA behaviors [14]. Parents can influence their children’s PA in a range of ways, including the provision of logistical and social support (e.g., transportation to training and encouragement), co-participation (i.e., participating together), and role modeling an active lifestyle [15–17]. A synthesis of results from 39 high-quality reviews [18] provided strong evidence that parents play a crucial role in promoting PA for children across various community settings (i.e., family and home, childcare, school). Given the role parents play in their children's PA engagement, parent direct involvement was a key element in designing the family-based intervention [19].

In response, researchers have designed and evaluated a range of PA interventions to improve PA and related physical and mental health development in the family context [19–21]. A systematic review reaffirms the crucial role that parents play in supporting and controlling children’s PA levels and found that both physical and social environmental factors operating within the home environment are important influences on school primary children’s PA patterns [22]. As such, changing the home physical environment and parenting behaviors are crucial in shaping and promoting children’s PA [23]. Lloyd et al. [23] conducted a randomized controlled trial to examine potential parenting behaviors of children’s PA change in the community program, and the findings indicated that the program positively impacts fathers' modeling of PA and parent–child co-PA.

However, family-based interventions showed mixed effects; some of them showed small-to-moderate effects or no effects [24, 25]. Findings from a systematic review and meta-analysis suggest that interventions with the involvement of parents to promote PA in children are effective, with 66% demonstrating a positive effect on PA [19]. Especially, parent-focused interventions involving the education of parents have previously been used to improve children’s PA behaviors. However, the level of parent involvement varied among previous research, and intervention design in which parents were targeted, while children were only involved in the data collection stage was still lacking in existing studies [26]. Accordingly, there is a need to provide more novel, effective, and feasible parent-focused interventions, especially in the current pandemic context. A negative consequence of stay-at-home orders and social distancing regulations was decreased opportunities for both parents and children to be physically active under the influence of COVID-19 [27, 28]. Particularly, schools and many workplaces were closed, and organized PA sessions were canceled in schools, resulting in transitioning activities that once took place outside of the home to online implementation in the home environment [29].

Parental involvement with children’s PL journey is considered crucial and meaningful, which is essential in supporting their child and their own PL development [30]. Lane et al. [30] designed a non-randomized, one-arm concurrent nested study to explore the feasibility of a PL program designed to build parental self-efficacy to support their child’s PL. The program shows promise as a PL intervention is feasible to influence parenting practices with regard to PL to develop children’s PL and PA. However, the study only included a small group of participants (35 parents of young children) and lack of a control group. Thereby, there is insufficient evidence to support the feasibility and effectiveness of delivery in an online format for interventions based on the PL framework.

Regarding intervention components, researchers have focused on different parts of PL to design and implement the intervention. Ha et al. [20] delivered ten face-to-face workshops and activity sessions to parents and children in a 6-month period in primary schools in Hong Kong to enhance children’s fundamental movement skill (FMS) competence and PA. For improving children’s PA participation and parents’ perceived motor competence, motivation, and knowledge about PA, Morgan et al. [21, 31] invited parents and children to attend seven face-to-face group sessions (90 min each) in a 3-month program in Australian school halls. Similarly, these two studies targeted different elements of PL framework to conduct the interventions through a series of organized face-to-face initiatives. Nevertheless, a recent systematic review reported that only 38.6% of the interventions formulated intervention components to cover all the core PL domains [32], and only a few researchers have explored the feasibility of interventions designed to improve PA in both children and parents with the mixed results, leading to insufficient understanding of “what PL-supportive programs look like in practice” [33]. Therefore, interventions are needed on committing to systematically address all domains of PL simultaneously [32].

### Current study

In Hong Kong, the rate of inactivity in children is high, and also in parents [34]. Referring to the conceptual framework proposed by Morgan and colleagues, which is an approach to the design and delivery of health behavior interventions targeting PA promotion, consists of four core intervention components: content, format, facilitator, and pedagogy. In terms of the content, reciprocal reinforcement was proposed, in which can be found when parents and children are independently encouraged to role model healthy behaviors at home [35]. Thus, increasing the activity levels of children and their parents may result in sustained changes in both parties, increasing the long-term effectiveness of the intervention. In light of the research gaps in the extant studies, in the current study, we examined the feasibility and effectiveness of a parent-led intervention that was designed to increase parent self-efficacy on PL and PA participation of primary school children and their parents. Our intervention was guided by the Whitehead's PL framework [9], which is considered a holistic and dynamic framework and is described as the motivation, confidence, physical competence, knowledge, and understanding that provides individuals with the movement foundation for lifelong participation in PA across multiple levels of the program design.

In this study, we conducted a non-randomized controlled trial to examine the feasibility and effectiveness of the intervention. In terms of feasibility, we used Bowen's framework [36], which is a conceptual framework that guides investigators on how to design a feasibility intervention. We focused on three areas to examine the intervention’s feasibility: acceptability, fidelity, and implementation. Concerning the effectiveness of the intervention, we hypothesized that participants in the experimental group, compared to those in the comparison group, would demonstrate larger improvements in PA, parent–child co-activity, family PA routines, and parent PL after receiving the 3-month intervention. To examine the effect of the intervention, all outcomes were measured at baseline and after the intervention.

## Methods

### Study design

We conducted a non-randomized controlled trial with one experimental and one comparison group. The protocol of the study was reviewed and approved by the university ethical review committee and prospectively registered at the Chinese Clinical Trial Registry (registration number: ChiCTR2100041903) on 09 January 2021. All methods were carried out in relevance with relevant guidelines.

### Sample size calculation

A power calculation was conducted to estimate the required sample size and the number of families based on the research questions. Calculations were conducted using G*Power 3.1.9.4, with an alpha level set to 0.05 and power to 0.8. Based on previous findings, the expected effect size was set at 0.5 [20]. The total required sample size was calculated to be 128 parent–child pairs. Conservatively, a 20% drop-out rate was estimated from baseline to post-intervention; a total of 160 parents and children was calculated to target as the recruitment sample size. To ensure the representativeness of the sample, we recruited participants from different districts in Hong Kong.

### Participants and recruitment

Participants in the study were a subsample of families who have consented to take part in the Fun to Move@JC project [34]. Fun to Move@JC is a school-based multi-component project launched in 2017 with the aim of enhancing the PA of primary school students and their parents. Participants of this project received a pair (one for the student and one for a parent) of wrist-worn PA trackers free of charge. They could also sign up for the project’s extra-curricular activity classes for free. Project schools received support from the project team to create or modify activity-friendly environments for students. Teachers from participating schools also received regular professional development training led by the project team.

We contacted teachers in the project schools (a total of 35 primary schools), asked them to help publicize the recruitment information for the current program. We also posted relevant information on the online platform to recruit participants. In January 2021, participants from 33 local primary schools in Hong Kong responded to our invitation. Parents and children who were from all three districts in Hong Kong (New Territories, Hong Kong Island, and Kowloon) volunteered to sign up to participate in our program.

In the current study, primary school children (i.e., Grade 1 to 6, typically aged 7–12 years) and their father or mother (grandparents or caretakers were excluded) were included. Children and parents with physical or mental disabilities that may influence their PA behaviors and those who are unable to understand and speak Cantonese and/or Mandarin were excluded from the study. Eligible parents received an invitation letter, which introduced the purpose of the study and the intervention components. An online briefing session was conducted to introduce the study design. Parents who agreed to take part in the program returned signed consent forms prior to baseline data collection. In order to establish whether the intervention was feasible and any changes in the outcome could be attributed to intervention components of the sub-study, but not to the parent project, data from families who did not enroll in the current study but participated in the Fun to Move@JC project were used for comparison purposes. These families were invited to complete surveys sent to them via a mobile application developed by the Fun to Move@JC project. Families who provided matching responses at the corresponding time periods as the intervention group was considered the comparison sample.

### Intervention

Guided by the evidence-based behavior change technique that was proposed by Michie et al. [37], the intervention included a series of interactive workshops for parents with activity sessions and PL homework with an informative take-home message. The intervention period lasted three months (between March and June 2021).

The intervention consisted of six 60-min parent workshops that were delivered online. The key topics and descriptions of the workshops are also shown in Table 1, and the workshops were delivered in the same order as they are presented in the table. The aims of the workshops were to 1) provide the informative message to enhance parents’ awareness of PA participation, self-efficacy on PA, and knowledge of PL; and 2) deliver playful activities, resources and instruct FMS that could increase parents’ motivation and competence in supporting their children's PL development and align with co-engagement in PA with children. Based on the elements of the behavior change technique, this was an example of how we operationalized it. For instance, one of the workshops is focusing on time management, the role of parents, and reducing screen time. We used the four behavioral change techniques. With regard to goals and planning, we suggest parents set a goal defined in terms of the behavior to be achieved and with a detailed action plan. We encourage parents to manage family PA time and establish a regular PA routine. To emphasize the role of parents, we used feedback and monitoring techniques to encourage parents' monitoring of behavior and provide informative feedback. We informed parents of the WHO PA guidelines and encouraged them to limit their children’s screen time. For the natural consequences, we provided information about health consequences of the behavior and emphasized the consequences of the behavior with the aim of making them more memorable. In terms of repetition and substitution, we focused on behavior substitution (e.g., suggesting that the person go for exercise rather than watching television in their leisure time) and habit formation (e.g., prompt repetition of the PA behavior on a regular basis until they form a regular PA pattern and a lifelong PL journey).

**Table 1:** Description of strategies mapped to the parental workshop

Workshop theme	Key concepts and activities	Behavioral change techniques	Domains of physical literacy
Time management, the role of parents, reduce the screen time	-Definition of “PA” and “MVPA” -PA prevalence and trends and importance of PA -Introducing the PA guideline (child and adult) -Role of parents on PA (support, encouragement, and involvement) -Time management & Goal setting -Formulating family routines, including PA and Co-PA routines -Suggestions and tips to parent–child co-activity (increase opportunities for PA) -Reduce recreational screen-time ***Activity:*** PA while housekeeping (introduce six activities while housekeeping, e.g., deep squat when sweeping the floor)	-1. Goals and planning -2. Feedback and monitoring -5. Natural consequences -8. Repetition and substitution	-Self-concept and affect -Motivation/Confidence -Social/experiential -Knowledge and understanding
Quality physical activity experience	-Importance of quality of physical education Role of the family in physical education (Parents can also be coaches for their children) Strengthen parents' awareness of physical education and PA Quality physical education is more than just fun; however, it is also a crucial academic discipline in primary school-aged children ***Activity:*** -Balance game (Coordination ability; Static balance; Dynamic balance; Cardiorespiratory Endurance)	-3. Social support -12. Antecedents	-Knowledge and understanding -Self-concept and affect -Social/experiential
Health-related fitness	-Definition of Health-related fitness and clarifications of fitness -Fitness prevalence and trends and importance of fitness for various academic and health outcomes -Highlight the Health-related fitness with examples: Flexibility & Cardiorespiratory Endurance & Muscular strength and endurance & Body composition -The concept of FITT (frequency, intensity, type, and time) -Assessing and training for health-related fitness -WHO recommendations on the intensity of the fitness ***Activity:*** -Fitness dice game including four domains: Flexibility, Cardiorespiratory Endurance, Muscular strength and endurance, and Balance	-2. Feedback and monitoring -4. Shaping knowledge	-Physical/Motor competence -Motivation/Confidence -Knowledge and understanding
Motivation & Enjoyment in PA	-Recap the critical points of the previous three workshops and sharing completed worksheets as examples -Underline the spirit of the “Victory without arrogance, defeat without discouragement” in the sport participation and daily lives -Improve exercise motivation, especially the intrinsic motivation -Self-management strategies and how to face the failure -Whole-person development through PA and sport ***Activity:*** -Stretching exercises	-4. Shaping knowledge -5. Natural consequences	-Motivation/Confidence -Enjoyment -Self-concept and affect -Social/experiential -Knowledge and understanding
Fundamental movement skills	-The importance of FMS: building blocks -Skill types: locomotor skills, ball skills, stability -Skill transfer: from FMS to sports ***Activity:*** -Movement skill teaching -Highlighting critical elements of each skill to instruct the FMS	-4. Shaping knowledge -5. Natural consequences	-Physical/Motor competence -Motivation/Confidence -Knowledge and understanding
Physical literacy	-Reinforcing the key messages of the six workshops -Key concepts of PL (i.e., I know, I can, I want, I do) -Linking PL to contents of previous workshops -Development of PL and sustaining behavior **Activity:** -The PL “North-East-South-West” game (note: the “North-East-South-West” is a traditional game that involves folding a square sheet of paper into a specific shape) **Graduation ceremony:** -summarizing the key points of the overall workshops -announcing parent ambassadors -parent sharing sessions (share the learning experience and suggestions) -all participating families were commended with certificates and gifts	-4. Shaping knowledge -7. Associations	-Self-concept and affect -Motivation/Confidence -Knowledge and understanding -Social/experiential

Furthermore, the intervention design was based on the holistic physical literacy framework focusing on systematically employ four domains of PL. Namely, affective domain (motivation and competence), physical domain (physical competence), cognitive domain (knowledge and understanding), and behavioral domain (engagement in physical activities for life). By combining theory with practice, parents can not only understand the knowledge and information related to physical literacy but also deepen their understanding of activities and transfer the knowledge to their future daily lives.

Workshops were delivered in real-time online due to the COVID-19 pandemic and to reach participants scattered in various districts in Hong Kong simultaneously. Parents who were in the experimental group attended the workshops biweekly via Zoom, a video conferencing service. Some parents reviewed the full workshop content with recorded videos if they were not able to attend the workshop through the live streams. The recorded videos were shared with parents after each workshop via WhatsApp (the most popular social networking application in Hong Kong). Each parent workshop was divided into two sections, a 20-min knowledge sharing and education session and a 40-min activity session. At the end of each workshop, parents were invited to share their insights and suggestions. The knowledge sharing and education sections in the workshops were delivered by the lead researcher, who had both theoretical knowledge and practice experience in the development of PL and PA interventions [20, 38, 39], parent education techniques [20], and community-based PA promotion [34]. Activity sessions were embedded in the workshops to enable learning and sharing in a more relaxed manner. Activity sessions for each topic were led by the coaches who held degrees in sports science or physical education. All the coaches have the basic knowledge and experience of PE teaching who led the interactive activity sessions to enable parents to understand the knowledge in practice. Parents learned movement patterns and exercises under the instruction and monitoring of the coach. Similar to in-person instruction, coaches provided key points to complete motor skill and encouraged parents to participate in the activity session. The workshop content was designed and developed by the lead researcher and research team using evidence-based behavior change techniques [37] and recommendations from previous literature in PL development [30, 40]. Furthermore, parents' preferences, suggestions, and other real needs were received via an online survey prior to the program's implementation. The workshops were tailored and developed to cater to the needs of parents.

Apart from intervention delivery via workshops, parents were given electronic worksheets after each workshop. The worksheets echoed the theme of the workshops and were designed for two objectives, namely self-learning and self-monitoring. Specifically, the worksheet covered the essence of each workshop for parents to review as a take-home message and encouraged parent–child PA, setting daily/weekly goals and reporting and reflecting on the relevant PA and/or FMS performed each day/week. Parents were asked to complete worksheets and submit them via WhatsApp.

In addition, to attract parents to attend the workshops, each workshop session was titled to a topic that is of interest to parents of primary school students. To avoid parents dropping out due to workshop contents being over technical or theoretical, workshop contents focused on practical tips on how parents can help children and themselves improve FMS and fitness, sustain PA behaviors, and, more generally in improve health and well-being. Besides, to enhance parent engagement and adherence, digital notifications and reminders were sent on a regular basis. After the intervention, in order to encourage and reinforce the knowledge and activities that parents have learned throughout the intervention and to apply and sustain the active routines in the future life, each participating family received a certificate, parent–child sports T-shirts, and portable exercise equipment to maintain active lifestyles and develop PL.

### Outcome measures

#### Physical activity of children and parents

Participants’ PA was measured using a PA tracker (referred to as the “sport band” within the project) developed by the project team. The sport band is a wrist-worn device that measures step counts, which is an accelerometer-based pedometer. Further, step counts were stored in 15-s epochs and then translated to measures of activity intensity for each interval. Data recorded from these devices were synchronized via mobile phone applications and/or through gateways placed in classrooms of students. In our pilot study, we found strong correlations between MVPA measured using ActiGraph wGT3X-BT accelerometers and Fun to Move@JC sport bands in children (*r* = 0.86) and parents (*r* = 0.87). Therefore, the sport band achieved satisfactory validity to measure the MVPA in both adults and children.

#### Frequency of parent–child co-activity

Parents completed the five-item questionnaire [41] for evaluating the frequency of parent–child co-activities and type of PAs. In the questionnaire, parents reported frequencies of five shared PA behaviors during leisure time with their children (i.e., walking or cycling, playing a sport/doing PA, performing outdoor activities in the park/playground, going to an indoor recreation center, and using active or inactive transport mode for a < 1 km short trips). The answers were recorded on a five-point Likert scale (never = 1, daily = 5).

#### Family physical activity routines

Family PA routines were measured using two parent-reported variables, namely PA routine and parent–child co-activity routine. Parents' responses to the following two questions were represented to the PA and Co-PA routine: “In your daily life, are there routines about PA participation in a typical week (1 = yes, 0 = no)?” and “In your daily life, are there routines about parent–child co-PA participation in a typical week (1 = yes, 0 = no)?” We developed the questions based on the previous research to understand whether family established given PA and Co-activity behaviors [42].

#### Perceived physical literacy

Perceived PL instrument was used to assess parents' PA knowledge, beliefs, and confidence [43]. The strength of the instrument allows for the exploration of in-depth responses around the cognitive domain of PL and for capturing social interactions with other individuals [44]. Three domains, with three items each, measured knowledge and understanding (“I am aware of the benefits of PA related to health”), self-expression and communication with others (“I have strong social skills”), and sense of self and self-confidence (“I am physically fit, in accordance with my age”). Parents were asked to indicate the extent to which they agreed with each statement on a 5-point Likert scale (1 = *strongly disagree*, 5 = *strongly agree*). Mean scores were generated for these three domains. All three domains achieved satisfactory reliabilities (Cronbach’s αs ≥ 0.85), the level of Cronbach’s alpha is acceptable [34].

### Feasibility measures

#### Workshop acceptability

After each workshop, parents were invited to evaluate the workshop. Specifically, parents responded to 6 Likert-scale questions (5-point; 1 = *strongly disagree,* 5 = *strongly agree)* regarding their overall opinion of the workshop, including if 1) they can master the contents; 2) the information they received from the workshop can meet their expectations; 3) the speaker's sharing was explicit; 4) the activity session was fun; 5) they can apply the knowledge they learned into the daily life; 6) the arrangement of the workshop was appropriate.

#### Program acceptability, fidelity, and implementation

Participants’ satisfaction and acceptability of the intervention with all intervention components were determined using a process evaluation questionnaire completed after post-intervention assessments. Besides, parents responded to two open-ended questions (“which part of the intervention were you most satisfied with”, and “suggestions and improvements to the intervention”) to indicate if the intervention was implemented as expected. To investigate the fidelity of the program, attendance of each workshop (including reviewing the recordings of all workshops) and submission of worksheets (on a bi-weekly basis) by parents were recorded. Taken together, 12 Likert-scale questions (5-point; 1 = *strongly disagree*, 5 = strongly agree) were designed to evaluate the acceptability, fidelity, and implementation of the program. This survey was delivered via the electronic form to parents one day after their attendance in the last workshop.

### Statistical analyses

IBM SPSS 24.0 (SPSS Inc, Illinois, USA) was used for all statistical analyses. To evaluate the intervention effects on measured outcomes, we followed the intention-to-treat principle for the participants. To deal with missing data, the last observation carried forward approach was used to combine the observed and imputed data then to analyze in order to conform to an intention to treat analysis [45]. We used random intercept, random slope linear mixed models to evaluate the effectiveness of the intervention between time, group, and groups over time. Specifically, the significance of the Time*Group terms in the regression models was examined. A significant Time*Group term would imply the existence of an intervention effect. The analyses were conducted for changes from baseline to post-intervention.

## Results

**Fig. 1:**
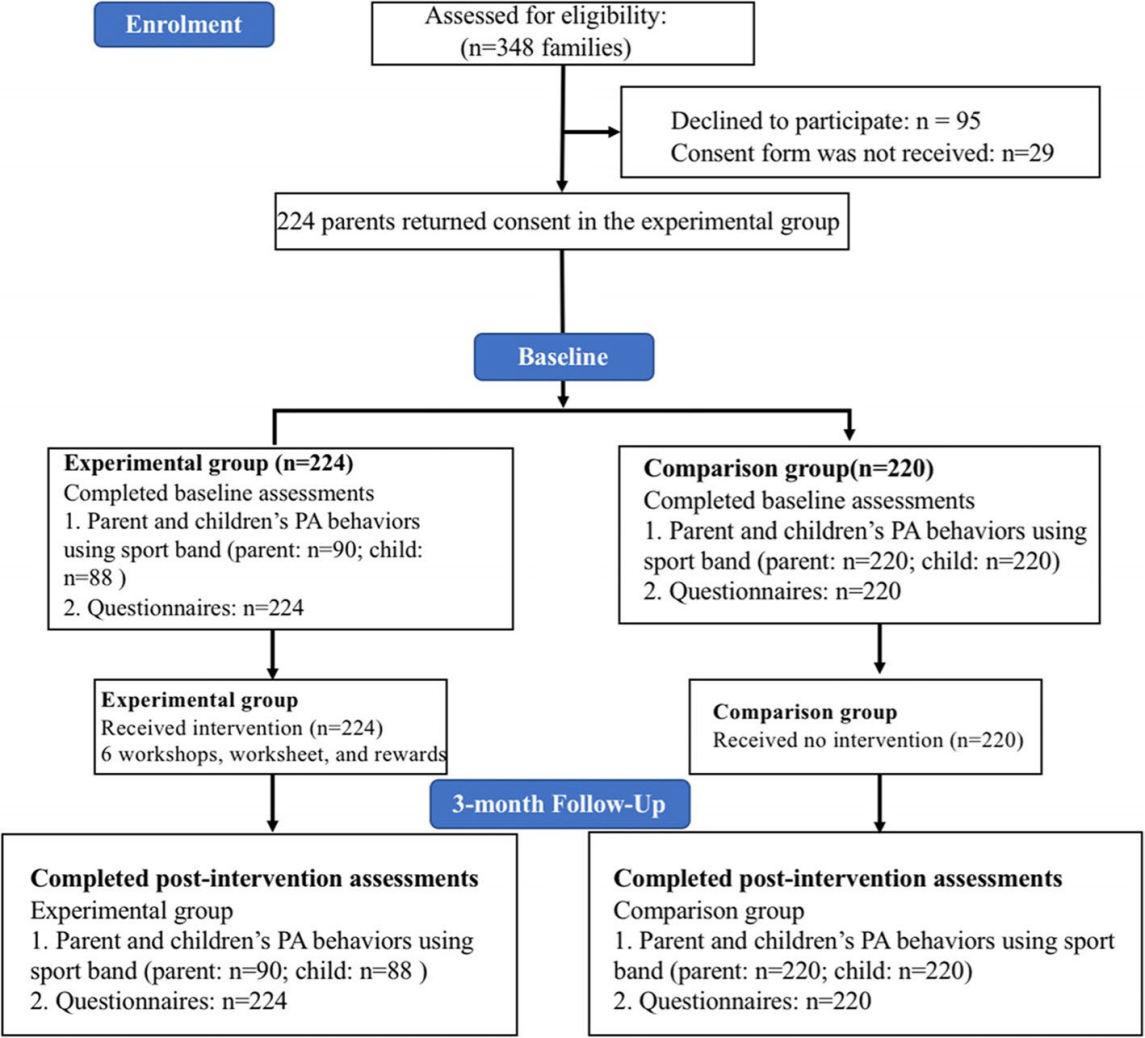
A flow diagram representing the non-randomized controlled feasibility trial

A total of 224 families, including 224 parents (85.7% of mothers and 14.3% of fathers, mean age = 39.73, *SD* = 8.14) and 224 children (58.9% of boys and 41.1% of girls, mean age = 9.28 years, *SD* = 1.80) from 33 Hong Kong primary schools returned the consent form and were considered the experimental group. In addition, 220 families who submitted data at the same time were considered the comparison group. Figure 1 presented the flow of participants through the intervention. Descriptive statistics in outcomes from baseline to post-intervention within intervention and comparison groups are displayed in Table 2. We examined the effect of the intervention on outcomes using linear mixed models (Table 3).

**Table 2:** Descriptive statistics of the measured outcomes at baseline and post-intervention for intervention and comparison groups

		**Baseline**						**Post-intervention**				
		**Intervention**			**Comparison**			**Intervention**			**Comparison**	
	**N**	**Mean**	**SD**	**N**	**Mean**	**SD**	**N**	**Mean**	**SD**	**N**	**Mean**	**SD**
Physical literacy	224	3.64	0.65	220	3.71	0.53	224	3.69	0.60	220	3.60	0.54
Co-PA routines	224	0.57	0.50	220	0.71	0.46	224	0.61	0.49	220	0.72	0.45
PA routines	224	0.45	0.50	220	0.49	0.50	224	0.49	0.50	220	0.53	0.50
Co-activity	224	3.50	0.65	220	3.47	0.58	224	3.54	0.66	220	3.33	0.59
Child steps (per day)	90	9,196.81	3,200.81	220	9,817.75	4,968.80	90	10,747.02	4,062.99	220	11,051.63	4,062.99
Child MVPA (min/day)	90	49.11	29.60	220	50.07	29.18	90	57.39	28.43	220	59.52	28.43
Parent steps (per day)	88	10,168.79	4,018.55	220	10,132.44	4,093.67	88	11,447.40	4,843.91	220	11,741.19	5,444.84
Parent MVPA (min/day)	88	61.38	30.16	220	62.56	32.19	88	68.74	32.60	220	72.87	37.25

Abbreviation: MVPA = Moderate-to-vigorous physical activity

**Table 3:** Effects of the intervention on the measured outcomes

	**Time**				**Group**				**Interaction**			
									Group*Time			
	**B**	**SE**	**(95%CI)**	***p***	**B**	**SE**	**(95%CI)**	***p***	**B**	**SE**	**(95%CI)**	***p***
Parent PL	-0.05	0.03	(-0.11,0.00)	0.067	-0.09	0.05	(-0.19,0.02)	0.100	0.16	0.04	(0.08,0.24)	< 0.001
Co-PA routine	-0.04	0.03	(-0.09,0.01)	0.114	0.11	0.04	(0.02,0.20)	0.018	0.03	0.04	(-0.05,0.11)	0.444
PA routines	-0.04	0.03	(-0.09,0.02)	0.211	0.04	0.05	(-0.05,0.13)	0.391	-0.01	0.04	(-0.09,0.08)	0.904
Co-activity	-0.04	0.03	(-0.10,0.02)	0.204	-0.20	0.06	(-0.32, -0.09)	0.001	0.17	0.05	(0.08,0.27)	< 0.001
Child steps (per day)	-620.94	539.43	(-1699.43,457.54)	0.254	1534.23	546.38	(453.17,2615.30)	0.006	316.33	637.60	(-943.30,1575.96)	0.621
Child MVPA (min/day)	-1.00	1.78	(-4.53,2.53)	0.575	9.49	3.66	(2.28,16.71)	0.010	-1.13	2.29	(-5.65,3.40)	0.623
Parent steps (per day)	40.68	289.25	(-533.83,615.19)	0.888	1614.00	598.85	(434.29,2793.71)	0.008	-334.46	385.77	(-1096.00,427.08)	0.387
Parent MVPA (min/day)	-1.18	1.79	(-4.72,2.36)	0.511	10.37	3.77	(2.95,17.78)	0.006	-2.95	2.34	(-7.54,1.64)	0.207

Abbreviation: *SE* Standard error, *CI* Confidence interval, *PL* Physical literacy, *MVPA* Moderate-to-vigorous physical activity,

Note: significance at *p* < 0.05

### Intervention effects on measured outcomes

We found no Time*Group interaction effects for parents’ daily steps (*B* = -334.46, 95% confidence interval (CI) [-1096.00, 427.08], *p* = 0.39) and time spent in MVPA (*B* = -2.95, 95% CI [-7.54, 1.64], *p* = 0.21). Moreover, children’s daily steps (*B* = 316.33, 95% CI [-943.30, 1575.96], *p* = 0.62) and MVPA (*B* = -1.13, 95% CI [-5.65, 3.40], *p* = 0.62) suggested no Time*Group interaction effects from Times 1 to 2.

A significant Time*Group effect in favor of the experimental group was found for parent–child co-activity from Times 1 to 2 (*B* = 0.17, 95% CI [0.08, 0.27], *p* < 0.001). In terms of family routines, no Time*Group interaction effects were found for PA routine (*B* = -0.01, 95% CI [-0.09, 0.08], *p* = 0.90) or Co-PA routine (*B* = 0.03, 95% CI [-0.05, 0.11], *p* = 0.44) from Times 1 to 2. With regards to parent perceived PL, the Time*Group interaction effect was significant in favor of the experimental group from Times 1 to 2 (*B* = 0.16, 95% CI [0.08, 0.24], *p* < 0.001).

### Workshop acceptability

The participating parents expressed relatively high satisfaction after they attended the workshops. Each question in each session scored more than 4 out of a total of 5 (Table 4). The average score for the acceptability of the workshop was 4.33. Parents were most satisfied with the speaker’s sharing, while they were least satisfied with the overall arrangement of the workshop.

**Table 4:** Acceptability of the workshop

Question	Workshop 1	Workshop 2	Workshop 3	Workshop 4	Workshop 5	Workshop 6
I can master the contents from the workshop	4.45 ± 0.70	4.29 ± 0.64	4.40 ± 0.63	4.40 ± 0.62	4.23 ± 0.60	4.27 ± 0.62
The information I received in this workshop can meet my expectations	4.28 ± 0.69	4.20 ± 0.71	4.35 ± 0.65	4.49 ± 0.63	4.39 ± 0.59	4.22 ± 0.68
The speaker’s sharing was clear	4.47 ± 0.57	4.47 ± 0.62	4.57 ± 0.63	4.38 ± 0.60	4.29 ± 0.66	4.41 ± 0.64
The activity session was fun and playful	4.18 ± 0.77	4.38 ± 0.63	4.38 ± 0.68	4.32 ± 0.62	4.16 ± 0.71	4.33 ± 0.74
I will apply the knowledge I learnt into the daily life	4.33 ± 0.82	4.36 ± 0.61	4.31 ± 0.65	4.45 ± 0.63	4.18 ± 0.64	4.19 ± 0.64
The arrangement of the workshop was appropriate	4.23 ± 0.72	4.34 ± 0.67	4.43 ± 0.67	4.29 ± 0.65	4.17 ± 0.62	4.25 ± 0.69

Note: Number of responses we received in each workshop: 1st = 60; 2nd = 89; 3rd = 81; 4th = 84; 5th = 79; 6th = 64

### Program acceptability, fidelity, and implementation

For the fidelity of the intervention, parents in the experimental group averaged an attendance rate of 73% for workshops and activity sessions. The parents who did not attend gave reasons for their absence including working overtime, staying home to care for younger children, illness, and time conflict. Parents who were absent from the workshops were provided with video clips of the workshops recorded. An average of 81% of parents reviewed the recordings after the workshops. In terms of the learning materials, we received 149 copies (i.e., 66.5% of parents submitted the worksheets) from the parents and provided individual feedback and encouragement to parents. Parents also shared photos and videos about home-based/outdoor activities via WhatsApp.

With regard to the acceptability of the program, participants provided positive feedback (i.e., score of above 3.5) on all intervention components (Table 5). One hundred fifty-two participants responded to questions about intervention acceptability. Their satisfaction with the whole program was evaluated from the arrangement, schedule, contents, information, learning materials, and mode. Moreover, 152 parents responded to the psychological and behavioral change after the intervention. Namely, knowledge of PA and PL, proficiency of FMS, motivation to participate PA, and frequency of co-participation in PA with their children. Of these, the worksheet was the most feasible as one component of the intervention.

**Table 5:** Feasibility of the intervention components

Components of the intervention	Mean	SD
The overall arrangement of parental workshops was reasonable	3.77	0.76
The overall content of the workshops was interesting	3.82	0.77
Parental workshops were rich in content	3.86	0.78
I enjoyed the mode of online learning, including the exercise experience	3.66	0.94
I found the knowledge learned from the workshops are useful	3.91	0.72
I found the worksheet is valuable and informative	3.93	0.75
I am satisfied with the program	3.93	0.77
I will recommend the workshop to other parents	4.22	0.68
I gained some knowledge about physical literacy and physical activity after the program	3.89	0.77
I mastered the competence of fundamental movement skills from the program	3.91	0.79
I became more motivated to participate in physical activity with my children after the program	3.84	0.79
Frequency for parent–child activities/sports were increased in the daily life after the program	3.87	0.83

Moreover, participants responded to two open-ended questions regarding their experience and suggestions to the program. With regard to the question of “which part of the workshop you are most satisfied with”, the majority of parents (64.47%, 98 out of 152) were most satisfied with the coaches. Besides, parents expressed their satisfaction with the interactive activities (59.87%, 91 out of 152), the speaker's sharing (54.61%, 83 out of 152), and the online delivery mode (47.37%, 72 out of 152). Furthermore, 70 parents provided feedback regarding the suggestions for program improvements. Among them, 27 parents suggested that the workshops could be arranged on weekends and longer in the duration of each workshop. In addition, parents also expressed that although online workshops were flexible, they expected to attend face-to-face workshops, outdoor activities, and parent–child interactive activity sessions. Besides, parents commended that the learning materials could be shared through online platforms, which would be more interactive and convenient.

## Discussion

The aim of this study was to assess the feasibility and effectiveness of a parent-focused PL intervention. Overall results from the current study demonstrate that the intervention was feasible and acceptable in supporting parents to establish physically active routines in the family setting. In terms of effectiveness, there was not an effect of the intervention on children’s and parents’ PA behaviors. However, there was a treatment-by-time effect in favor of the experimental group was found for parent perceived PL and parent–child co-activity frequency from baseline to post-intervention.

With regard to the effectiveness of the intervention, no Time*Group interaction effect was found in PA among children and parents. Similar to previous findings, it is challenging to change parents’ and children’s MVPA in the short term through parent-focused interventions [20, 46, 47]. This might be attributed to a variety of factors. First, since the study used a non-randomized design, there may have been a healthy volunteer bias as participants across groups were generally already meeting MVPA recommendations at baseline, which could have resulted in ceiling effects. Thereby, excluding families that are sufficiently active or through randomization in an adequately powered trial could be considered in the future [47]. Furthermore, the intervention focuses on parent education, intending to help parents understand what each component of PL entails and support children's PL development. Despite the significance of PA being emphasized, it is difficult for people to make a fundamental change from cognition to practice [48]. As such, researchers still need to carry out more novel explorations and attempts to bridge the intention-to-action gap, finding further ways to promote PA participation, such as regular family involvement in PA sessions, including prioritizing PA time in daily family routine, reinforcing the role of parents, etc.

In this study, a significant Time*Group effect in favor of the experimental group was found for parent-perceived PL. Our findings supported previous research findings [20, 30, 31, 49], indicating that parent education training sessions might have a favorable impact on parenting practices related to PL. PL development is a lifetime process that influences various health outcomes [8]. Parental education and training appear to have the potential to increase parents’ PL knowledge and self-efficacy, in turn, promoting their child’s development of PL. Parental engagement is crucial, which is achieved through continuous support of learning in the home environment by engaging their children in PL development (i.e., establishing family routines such as doing no less than one hour of PA per day, being physically active as a family). If parents were physically active and were motivated to do more physical activities with confidence after the intervention, they may have taken the initiative to provide support in the home environment to develop PL for their children. Additionally, parents may more likely be able to seek support or put their children in situations to develop their PL. Through this intervention, the PL of parents was improved and consciously carried out parent–child activities.

A positive intervention effect in favor of the experimental group was observed for parent–child co-participation in PA from baseline to post-intervention. In line with a previous systematic review [50], parents’ co-participation in PA with their children was crucial to promoting and sustaining PA behaviors in both parties. In the current intervention, goal setting and self-monitoring using worksheets provided encouraged parents to monitor and schedule their PA/co-PA behavior. The worksheets also included recommended activities that parents could do with their children together in their daily lives. Goal setting has demonstrated some success in adults [51] and helps in setting goals in motion following their values, priorities, and commitment to change [52]. Using behavior change techniques, such as ‘prompt specific goal setting’ [53], was considered to be an appropriate method to let parents make or support children’s behavior change. We received 149 copies of the worksheet in the current study (i.e., 66.5% of parents submitted the worksheets). Future research could consider designing the use of worksheets into interventions, including setting PA goals, recommending physical activities, and inviting parents to fill in their joint activities and feelings. This approach may help parents make PA a part of their family routine. Besides, the worksheets would reinforce parents’ understanding by “forcing” them to reflect on the workshop contents. What is particularly noteworthy is that researcher and practitioner could encourage parents to complete worksheets and gain insight into the reasons for their failure to do so, which could lead to behavioral changes, such as scheduling more PA and parent–child co-PA time. Furthermore, longer follow-up periods are required in future research to determine medium- to long-term behavior change and confirm these effectiveness results.

Several factors may have contributed to the high acceptability of the program reported by parents. First, prior to the program implementation, parents’ preferences, suggestions, and other real needs were received via an online survey. In response, the informative and fruitful contents of the parental workshops and activity sessions were tailored and developed. Information we found from the open-ended responses in the process evaluation survey, several parents commented that this was the first time they could access evidence-based information and fun activities to understand the importance of PL. The high parental ratings of the workshops were revealed, supporting the ‘train the trainer’ approach [11] used in the home environment to improve PA levels and PL development for both parties. Third, for most parents, online learning is a viable option. Using online mode to deliver the intervention can avoid direct interaction between persons under the impact of the pandemic. Furthermore, for a majority of parents, compared with face-to-face participation, online learning is very adaptable, flexible, easy, and convenient. Consistent with the findings in other online PA interventions [47, 54], online education and learning are acceptable in adults and have the potential to be important in the global pandemic of physical inactivity. This parent-focused program, delivered online in the context of the COVID-19 pandemic, provides some theoretical and practical contributions to interventions designed with the PL framework. Practically, the online workshop sessions can provide a vehicle for parents and children who have been deprived of physical activities as a result of the pandemic’s social distancing regulations.

There are some notable strengths in the intervention. First, each parent workshop consists of parent education and activities, and the delivered contents combined scientific with practical perspectives. University academics (i.e., lead author) served as the speaker of parent education sessions, who is more trustworthy to parents. Parents think they generally lack theoretical knowledge, and they will have more confidence and trust from more authoritative figures, such as university professors. In addition, interactive activity sessions led by research team members were also well received by parents. The interactive activity session covers FMS, physical fitness, home-based physical activities, and parent–child activities. Besides, coaches were trained to use the SAAFE (i.e., supportive, autonomous, active, fair, enjoyable) principles [40] in their instruction and interaction to meet the requirements of participants. Second, to ensure the intervention fidelity, we also communicated with coaches and provided timely feedback to ensure that all sessions were carried out as intended. Third, all the workshops were delivered by online training mode, which is highly accepted by parents. This mode is promising while maintaining social distancing and making home exercise possible in the current epidemic situation. Even after the COVID-19 pandemic, online delivery presents an opportunity to widen access to certain groups of people.

Despite its strengths, the limitations of the intervention should also be recognized. First, the current intervention was designed to be relatively short-term research. As a further direction, a longer-term follow-up test to examine the sustainability effects is needed. Second, the number of fathers involved in the study is relatively low. Although fathers were encouraged to sign up during program recruitment, given the different parenting styles and nature of employment of fathers and mothers, they may play different roles in the growth and development of children. Thus, more fathers should be recruited for future studies. Third, physically active parents were more likely to participate in the study. Besides, the compliance rate of PA should be further improved. Future studies are needed to identify ways to recruit parents who are less physically active in order to obtain a more representative sample and provide tangible and intangible incentives to participants who have valid PA data and completed the whole data collection procedure. Fourth, future research can measure additional outcomes that are related to PL, such as objectively measured FMS and physical competence, motivation, self-efficacy, and other parenting practices. This may allow us to have a more comprehensive understanding of whether the PL of parents and children could be changed through the intervention. Lastly, self-reported methods may have some bias in that parents tend to provide socially desirable responses. Qualitative research can be carried out to provide more in-depth information.

## Conclusions

In conclusion, this parent-focused intervention was based on the holistic PL framework focusing on employing four elements of PL systematically to design and implement the intervention with encouraging direct parental involvement in supporting PA participation in children and PL development in parents, which yielded some positive results. The program represents a novel venture that resulted in significant improvements in parent perceived PL and frequency of co-activity, yet more research is needed to explore how similar parent-focused PL interventions could produce changes in PA behaviors among children and parents. Since the intervention was delivered online, it has the potential to be scaled-up to reach a wider audience, the online modes allow us to include more of an audience from a wide geographic location at the same time. A hybrid format, namely combining face-to-face activity sessions with online education sessions, might be possible when the pandemic is over. The ultimate intention of the intervention is to appeal and involve more parents in paying more attention to PL development, which would, in turn, have positive health benefits for families. Future implementation at a larger scale should be appropriately powered and adopt randomization to minimize sampling bias, enhance generalizability, and elucidate longer-term outcomes.

## Data Availability

The datasets used and/or analyzed during the current study are available from the corresponding author on reasonable request.

## Abbreviations

PL: Physical literacy
PA: Physical activity
MVPA: Moderate-to-vigorous physical activity
FMS: Fundamental movement skills
SAAFE: Supportive, Active, Autonomous, Fair, and Enjoyable
